# 

**Published:** 1920-12-11

**Authors:** 


### Personalia.

After twenty-four years Mr. J. Taylor, J.P., O.B.E., has resigned the post of Chairman to the Working-men's Committee of the Guest Hospital, Dudley. The new Chairman is Mr. F. J. Ballard.

Dr. J. C. Marsh, Medical Officer to the Yeovil Board of Guardians, recently resigned after forty years' service.

The Derby Mental Hospital, in its latest report, mentions with regret the loss sustained by the institution through the retirement of Dr. S. R. Macphail, who has been its medical superintendent for thirty-two years.

### The Book Market.

Geo. Allen and Unwin, Ltd.: "The State and Sexual Morality." 1s. 6d. net.

Oliver and Boyd, Edinburgh: "Income Tax and Super Tax, 1842-1921." 1s. net.

John Wright and Sons, Ltd., Bristol: "A Synopsis of Medicine." By H. L. Tidy, M.A., M.D., B.Ch., F.R.C.P. 26s. net, post free.

Liverpool Booksellers Co., Ltd., and Simpkin, Marshall, Hamilton, Kent and Co., Ltd.: "Hypnotism and Treatment by Suggestion." By A. E. Davis, F.R.C.S., L.R.C.P. Third edition. 5s. net.

G. P. Putnam's Sons: "The Control of Parenthood." By James Marchant, C.B.E., LL.D., F.L.S. 7s. 6d. net.

Cassell and Co., Ltd.: "Clinical Methods" (seventh edition). By R. Hutchison, M.D., F.R.C.P., and H. Rainy, M.D., F.R.C.P., F.R.S.E. 12s. 6d. net.

J. Bale, Sons and Danielsson, Ltd.: Synoptic Chart of Cardiac Examination. Arranged by J. D. Comrie, M.A., B.Sc., M.D., F.R.C.P.E. 4s. 6d. net.

The Universal Medical Periodicals, Ltd.: "Intestinal Disinfection." By J. T. Ainslie Walker, Captain R.A.M.C. (T.F.). 6d. net.

### Matrons' and Sisters' Appointments.

Victoria Hospital for Children, Chelsea.—Miss Jessie Smales has been appointed matron. She was trained at King's College Hospital, was for three years assistant matron of the Queen's Hospital for Children, and since June 1919 has been matron of the Kent and Canterbury Hospital.

Uxbridge Union Infirmary, Hillingdon.—Miss Daisy Watson and Miss Edith Heap have been appointed sisters. Miss Watson was trained at Norwich Infirmary, and Miss Heap at St. Mary's (Islington) Infirmary and the East End Mothers' Home.

General Hospital, Malvern.—Miss Cornish and Miss Mary Ball have been appointed sisters. They were both trained at Addenbrooke's Hospital, Cambridge.

Samaritan Hospital for Women, Belfast.—Miss Lois Oakes has been appointed sister. She was trained at the Princess Christian Hospital, Weymouth, and at the Rotunda Hospital, Dublin; she has held the post of theatre, ward, and night sister at the former institution.

Cape Maternity Hospital, Capetown.—Miss Teresa R. Mullan has been appointed matron. She was trained at the Royal Southern Hospital, Liverpool, and at the Dundee Maternity Hospital, and has since been theatre sister and assistant matron at the Rotunda Hospital, Dublin.

